# Human Chorionic Gonadotropin (hCG)—An Endocrine, Regulator of Gestation and Cancer

**DOI:** 10.3390/ijms19051502

**Published:** 2018-05-17

**Authors:** Helene Heidegger, Udo Jeschke

**Affiliations:** Department of Obsterics and Gynecology, University Hospital, LMU Munich, 81377 Munich, Germany; Helene.heidegger@med.uni-muenchen.de

Human Chorionic Gonadotropin (hCG) is a heterodimeric glycoprotein composed of two subunits [1]. This important and very complex molecule, that exists in different molecular forms, is implicated in all major reproductive and developmental processes in humans [1,2]. Although the title implies that hCG is an endocrine regulator, we are aware that hCG actions during pregnancy (except luteal regulation) and in cancers are largely paracrine and/or autocrine in nature. In the last years, the significance and the importance of hCG have really expanded, and many studies suggest an important role of this glycoprotein in the field of pregnancy. 

hCG is secreted by the syncytiotrophoblast which originates from fused and differentiated cytotrophoblast cells [3,4]. For a long time, the main known role of hCG was the promotion of progesterone secretion by the corpus luteum in early pregnancy, acting via the hCG/LH (luteinizing hormone) receptor. However, more recently, many other functions of hCG, not only in the placenta but also in the myometrium, the uterus, and the fetus, have been described [5,6,7]. 

In addition, the LH/hCG receptor is also expressed on granulosa cells. Casarini et al. have reported that the signaling pathways of hCG and LH do not completely overlap, and this fact may have implications for hCG use in assisted reproductive techniques (ART) [8]. Hershko Klement et al. described that a gonadotropin-releasing hormone (GnRH) agonist, initially presented as a substitute for hCG, has led to a new era of administering a GnRH agonist followed by hCG triggering [9].

hCG promotes angiogenesis and vascular genesis in the uterine vasculature during pregnancy [10,11], whereas its role in placental growth and development is incontrovertible. The hCG/LH receptor was also found in fetal organs, and thus it is suggested that hCG plays an important role in organ growth and differentiation in the fetus [12,13,14]. A function for hCG in umbilical cord growth and development has also been reported in the literature [15]. Many additional different immunomodulatory effects of hCG are described [16,17,18]. 

Schumacher et al. found that hCG determines fetal fate by regulating maternal innate and adaptive immune responses, allowing the acceptance of the foreign fetal antigens [19]. Environmental pollution can disturb hCG function during pregnancy. Paulescu et al. showed that prenatal exposure to selected endocrine-disrupting chemicals like Bisphenol A can have a deleterious impact on the fetus and long-lasting consequences also in adult life [2]. 

Some new data have shown a function of hCG in the implantation process [20,21]. Makrigiannakis et al. showed that hCG is one of the key molecules during the process of implantation. hCG effectively modulates several metabolic pathways within the decidua, contributing to endometrial receptivity [22]. In addition, the hCG/LH receptor has also been identified in adults women’s brain, a finding that could explain the hyperemesis gravidarum during pregnancy [23]. 

The expression of hCG is observed in several types of malignancies, including prostate cancer [24], colorectal cancer [25], lung adenocarcinoma [26], and different gynecological cancers, such as endometrial adenocarcinoma, breast cancer [27], cervical carcinoma [28], and ovarian cancer [29], and is associated with especially poorly differentiated and high-grade tumors [30]. Human chorionic gonadotrophin may be also a possible mediator of leiomyoma growth during pregnancy [31].

Finally, Theofanakis et al. showed that hCG could have a potential role as an anti-rejection agent in solid organ transplantation [32].

In summary, hCG is a multifaceted hormone with a very wide range of actions. In this Special Issue, “hCG—An Endocrine, Regulator of Gestation and Cancer” we have tried to highlight the different functional aspects of hCG and give a promising insight into different pathophysiological aspects, clinical applications, and therapeutic options related to hCG. 
